# GraphTeams: a method for discovering spatial gene clusters in Hi-C sequencing data

**DOI:** 10.1186/s12864-018-4622-0

**Published:** 2018-05-08

**Authors:** Tizian Schulz, Jens Stoye, Daniel Doerr

**Affiliations:** 1 Faculty of Technology and CeBiTec, Bielefeld University, Universitätsstr. 25, Bielefeld, 33615 Germany; 2 International Research Training Group 1906 “Computational Methods for the Analysis of the Diversity and Dynamics of Genomes”, Universitätsstr. 25, Bielefeld, 33615 Germany

**Keywords:** Spatial gene cluster, Gene teams, Single-linkage clustering, Graph teams, Hi-C data

## Abstract

**Background:**

*Hi-C sequencing* offers novel, cost-effective means to study the spatial conformation of chromosomes. We use data obtained from Hi-C experiments to provide new evidence for the existence of *spatial gene clusters*. These are sets of genes with associated functionality that exhibit close proximity to each other in the spatial conformation of chromosomes across several related species.

**Results:**

We present the first gene cluster model capable of handling spatial data. Our model generalizes a popular computational model for gene cluster prediction, called *δ-teams*, from sequences to graphs. Following previous lines of research, we subsequently extend our model to allow for several vertices being associated with the same label. The model, called *δ-teams with families*, is particular suitable for our application as it enables handling of gene duplicates. We develop algorithmic solutions for both models. We implemented the algorithm for discovering *δ*-teams with families and integrated it into a fully automated workflow for discovering gene clusters in Hi-C data, called GraphTeams. We applied it to human and mouse data to find intra- and interchromosomal gene cluster candidates. The results include intrachromosomal clusters that seem to exhibit a closer proximity in space than on their chromosomal DNA sequence. We further discovered interchromosomal gene clusters that contain genes from different chromosomes within the human genome, but are located on a single chromosome in mouse.

**Conclusions:**

By identifying *δ*-teams with families, we provide a flexible model to discover gene cluster candidates in Hi-C data. Our analysis of Hi-C data from human and mouse reveals several known gene clusters (thus validating our approach), but also few sparsely studied or possibly unknown gene cluster candidates that could be the source of further experimental investigations.

## Background

Distance-based clustering algorithms are paramount to approach various questions across all data-driven fields including comparative genomics. Here, we study the problem of discovering single-linkage clusters of a set of corresponding vertices (where correspondence between vertices across graphs is either provided through a bijective mapping or equivalence classes) between two or more undirected weighted graphs *G*_1_,…,*G*_*k*_ such that the weakest link in the cluster (measured in terms of the weighted shortest path) does not exceed a given threshold *δ* in either graph *G*_*i*_, 1≤*i*≤*k*. We call such clusters(*δ*-)teams, thereby adopting notation used by an extensive trail of literature that studies the equivalent problem on permutations and sequences [1–4].

**Fig. 1:**
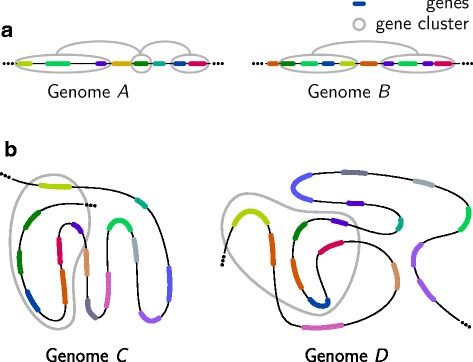
Illustrations of **a** sequential and **b** spatial gene clusters. Genes with the same colors belong to the same gene family

A prominent use case of *δ*-teams in comparative genomics is the detection of *gene clusters*, which are sets of genes with associated functionality such as the encoding of different enzymes used in the same metabolic pathway. In many organisms, instances exist where such genes are also locally close to each other in the genome, i.e., their positions fall within a narrow region on the same chromosome. They may even remain in close proximity over a longer evolutionary period, despite the fact that genomes regularly undergo mutations such as genome rearrangements, gene- or segmental duplications, as well as gene insertions and deletions. Such mutations may also affect the order and copy number of genes within a gene cluster (see example in Fig. 1a). Molecular biologists argue that a conserved neighborhood is beneficial for co-regulation, as is true in the prominent case of *operons* in prokaryotes [5]. Gene clusters are also prevalent in eukaryotes, even in animals, where the HOX gene cluster is without doubt the best studied representative. HOX genes are transcription factors that regulate the embryological development of the metazoan body plan [6].

Yet, the function of many genes is often barely understood or entirely unknown despite the increasing number of whole genome data that is becoming available. Hence, a popular approach in comparative genomics is to work this way backwards, starting with the investigation of conserved gene proximity in genomes of a reasonably phylogenetically diverse set of species. Here, the underlying assumption is made that accumulated genome rearrangements will have shuffled the genome sequences sufficiently so that natural selection becomes a plausible cause of conserved gene neighborhoods. By identifying homologous sets of genes that are consistently close to each other across several species, candidate gene clusters are identified that are then subject to more thorough functional analysis.

Recently, new technologies emerged, allowing the study of the spatial structure of genomes. *High-throughput chromosome conformation capture* (Hi-C), the most popular among such approaches, allows assessing the conformation of the chromatin structure in a cell sample through measuring the number of observed contacts between DNA regions [7]. The Hi-C method makes use of formaldehyde to covalently link proteins and DNA strings which are located next to each other in the cell. After crosslinking, the cells are lysed and the DNA is digested by a restriction enzyme. Remaining fragments bonded by the same protein are ligated. Sequencing the hybrid sequences reveals three-dimensional contacts between their genomic origins. The outcome of the experiment is a table, called *Hi-C map*, that records observed contacts either within a single (*intrachromosomal*) or between different chromosomes (*interchromosomal*). Each row and each column of the Hi-C map represents an equally sized segment of a genome sequence, and a count in each cell indicates how often hybrid sequences of the corresponding segments have been observed in the experiment. The size of these segments is known as *resolution*. It is a crucial parameter regarding the quality of the data. The higher the resolution of the chromatin structure is, the smaller is the segment size, but also the more data is needed to get significant results. An increasing number of Hi-C maps is made publicly available (human and mouse [8, 9], fruit fly [10]) and is used to answer numerous biological questions, starting from gene regulation and replication timing [8, 11] to genome scaffolding and haplotyping [12, 13].

Gene cluster discovery has sparked the development of various computational models for identifying sets of genes that exhibit close proximity. Such models typically rely on abstract data structures known as *gene order* sequences, which describe the succession of genes in chromosomes. In doing so, each element of a gene order sequence is the identifier of a gene’s associated gene family. A popular method to find gene clusters is based on the identification of *common intervals* in these sequences, which are intervals with an identical set of elements (i.e. gene family identifiers), independent of the elements’ order and multiplicity [14–16]. Since their first mentioning in [16], common intervals became the source for several generalizations [17, 18], among others, *δ-teams* [2]. *δ*-Teams are sets of elements where the distance between any two successors across all sequences is bounded by a given threshold *δ*≥0. This flexible model facilitates not only the detection of gene clusters that are interspersed by unrelated inserted genes, but also the consideration of general distance measures. The latter, for instance, allows to take into account the number of nucleotide base pairs between genes.

Not only the gene proximity on linear DNA sequences, but especially the spatial conformation of chromosomes may provide a pivotal indicator for common regulation and/or associated function of sets of genes (see example in Fig. 1b). Evidence of *spatial gene clusters* has been put forward already by Thévenin et al. [19] who studied spatial proximity within functional groups of genes in the human genome. In the present work, we introduce the first spatial gene cluster model. It extends the *δ*-teams model from sequences to undirected weighted graphs, facilitating the detection of genes that are consistently spatially close in multiple species. In doing so, our method integrates Hi-C and genome annotation data into weighted undirected graphs, where vertices represent gene family identifiers of genes and weighted edges correspond to distances obtained from Hi-C data.

The remainder of this manuscript is organized as follows: In the following section, we formally define *δ*-teams on graphs and present an algorithm for their discovery. We then extend our approach to finding *δ*-teams *with families*, i.e., the case where vertices across graphs are related through a common family membership, allowing multiple members of the same family to be part of the same graph. We then show how *δ*-teams can be used to find candidate sets of spatial gene clusters using a combination of genome and Hi-C data of two or more species. In “Results”, we present GraphTeams, a workflow for discovering gene cluster candidates in Hi-C data. We subsequently apply it to intra- and interchromosomal Hi-C data from human and mouse. Spatial gene clusters that have been found by our method are presented and further investigated. In “Discussion” we relate the computational complexity of our algorithms for finding *δ*-teams in graphs with those of permutations and sequences that have been previously reported in the literature. We then discuss some of the gene cluster candidates that our method discovered in intra- and interchromosomal Hi-C data of human and mouse. “Conclusions” closes this manuscript and provides an outlook on future work.

GraphTeams is available for download at http://github.com/danydoerr/GraphTeams.

## Methods

### Discovering *δ*-teams in graphs with shared vertex sets

In this section, we discuss the general problem of identifying common single-linkage clusters in a collection of graphs, where the largest link does not exceed a given distance threshold *δ*. We call such clusters *δ*-teams to remain in line with previous literature which studied the equivalent problem on permutations and sequences.

A naive method would require to compute all-pairs-shortest-paths in each graph independently. From these distances, a single matrix would be constructed for vertices that are common to all graphs. Each entry of this matrix, corresponding to a pair of vertices, equals to the longest distance over all shortest paths in any of the graphs. Then, a standard single-linkage clustering algorithm could be used to enumerate *δ*-teams. Here, we present an algorithm with a slightly better running time than this naive approach.

To simplify presentation, we describe only the case of two input graphs *G* and *H* in detail. The general case can be trivially inferred. In fact, our implementation (see “Results”) supports two or more graphs.

We study undirected graphs *G*=(*V*,*E*) with distances measure $d_{G}: V \times V \to \mathbb [0, \infty)$. While subsequent definitions adhere to the general case, for all our purposes we assume edge-weighted graphs and use as distance measure the length of the shortest path between any two vertices, if such exists and $\infty $ otherwise. In doing so, the length of the shortest path is measured by the sum of its edge weights. We use *E*(*G*) and *V*(*G*) to denote the edge and vertex set of a graph *G*, respectively. Since we will refer frequently to sets of vertices in one of several graphs, we will indicate the origin of a vertex set $S \subseteq V(G)$ of a graph *G* through subscript notation, i.e. *S*_*G*_, whenever this information is relevant. We are interested in sets of vertices that are connected through paths on which the distance between two successive members is bounded by *δ*:

#### **Definition 1**

[ *δ*-set] Given a graph *G* with distance measure *d*_*G*_ and a threshold value *δ*≥0, a vertex set $S \subseteq V(G)$ is a *δ-set* if for each pair of vertices *u*,*v*∈*S* there exists a sequence $P = (u, \ldots, v) \subseteq S$ such that the distance *d*_*G*_(*w*,*z*) between any two consecutive vertices *w* and *z* of *P* is less than or equal to *δ*.

Note that unlike single-linkage clusters of a graph, *δ*-sets are not required to be maximal in that graph. Analog to *partition refinement*, the aim is to find sets of vertices that are *δ*-sets in both input graphs. The subsequent definitions establish relations of *δ*-sets across two graphs *G* and *H* with shared vertex set $V_{\cap } = V(G) \cap V(H)$. In doing so, we assume that there is a common non-empty set of vertices between the two graphs that is subject to subsequent analysis. Vertices that are unique to either of the two graphs are disregarded, yet may be relevant due to their involvement in paths between common vertices.

#### **Definition 2**

[ *δ*-cluster] Given two graphs *G* and *H* with distance measure *d*_*G*_ and *d*_*H*_, respectively, and a threshold value *δ*≥0, a vertex set $S \subseteq V_{\cap }$ is a *δ-cluster* if it is a *δ*-set in both *G* and *H* under distance measures *d*_*G*_ and *d*_*H*_, respectively.

#### **Definition 3**

[ *δ*-team] Given two graphs *G* and *H*, a *δ*-cluster *S* of *G* and *H* is a *δ-team* if it is *maximal*, i.e., there is no *δ*-cluster *S*^′^ of *G* and *H* such that $S \subsetneqq S^{\prime }$.

#### **Example 1**

**Fig. 2:**
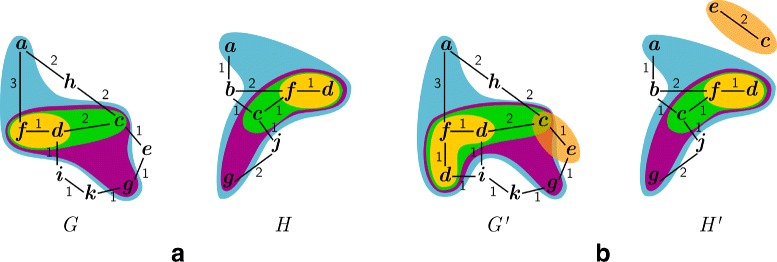
Examples of *δ*-teams and *δ*-clusters in graphs without families **a** and with families **b**. *δ*-Teams and -clusters are highlighted by areas of shared color. Edge labels indicate weights. Vertices in **b** are represented by their family identifier

The two graphs *G* and *H* depicted in Fig. *2*a have three *δ*-teams: 1-team {*d*,*f*}; *2*-team {*c*,*d*,*f*}, and *3*-team {*a*,*c*,*d*,*f*,*g*}. The set {*c*,*d*,*f*,*g*} exemplifies a non-maximal *3*-cluster of *G* and *H*.

### Finding *δ*-teams by decomposing graphs with divide-and-conquer

Given the above definitions, the following computational problem naturally arises and is subject to this work:

#### **Problem 1**

Given two graphs *G* and *H* with distance measure *d*_*G*_ and *d*_*H*_, respectively, and a threshold value *δ*≥0, find all *δ*-teams of *G* and *H*.

The first observation that is key to addressing the problem at hand, is that two *δ*-teams cannot overlap. The following lemma, in which Teams_*δ*_(*S*) denotes the set of *δ*-teams of vertex set *S*, is basis to all permutation-based (gene-) team algorithms and holds true for the here proposed generalization, too:

#### **Lemma 1**

*[*1*,*^2^*]* Given two graphs *G* and *H* with common vertex set $V_{\cap }$ and a threshold value *δ*≥0, there exists a partition $\{V^{\prime },{V^{\prime }{\prime }}\}$ of $V_{\cap }$ such that $\text {Teams}_{\delta }(V_{\cap }) = \text {Teams}_{\delta }(V^{\prime }) \cup \text {Teams}_{\delta }(V^{{\prime }{\prime }})$.

The lemma leads to a simple divide-and-conquer approach which has already been applied by He and Goldwasser [2] for the restricted case of sequential data. Here, we apply this lemma to general graphs. Algorithm DECOMPOSE divides the common vertex subset $S \subseteq V_{\cap }$ of graphs *G* and *H* into smaller subsets as long as *S* is not a *δ*-set in both graphs.



Because Algorithm 1 proceeds from larger to smaller sets, a vertex set *S*, identified by the algorithm, that is a *δ*-set in both *G* and *H* is always maximal and therefore a *δ*-team. Procedure SMALLMAX (see line 1) finds a maximal *δ*-set $S^{\prime }$ smaller than *S*, or, if the smallest maximal *δ*-set (that is still a subset of *S*) in both *G* and *H* is *S* itself, returns *S*. This will be further elaborated in the following section.

### Identifying maximal *δ*-sets

Maximal *δ*-sets are identified by function SMALLMAX as described in pseudo-code by Algorithm 2.



Note that procedure SMALLMAX is drafted for a general setting that permits the discovery of different vertex sets in graphs *G* and *H*, respectively. In doing so, SMALLMAX can also be used in the case of finding *δ*-teams *with families* that is subject of “*δ*-teams with families”. For now, the input sets *S*_*G*_ and *S*_*H*_ are identical.

The function is designed to terminate the search for a maximal *δ*-set that is subset of *S*_*G*_ or *S*_*H*_ as early as possible. Hence, SMALLMAX searches through both graphs independently, but simultaneously, to identify a smaller maximal *δ*-set in either vertex set. In each iteration (lines 4-12), the algorithm tries to identify in each graph *X*=*G*,*H* a vertex *s* of set *S*_*X*_ which has not been previously visited and that has distance at most *δ* from any already visited node. To this end, a list $S^{\prime }_{X}$ is maintained that keeps track of already visited vertices of set *S*_*X*_. Boolean variables *p*_*X*_ indicate whether unvisited, yet reachable vertices in set $S_{X} \setminus S^{\prime }_{X}$ could be found in graph *X* in the previous iteration. The iteration is controlled by three different cases (line 4): If no unvisited node can be found, SMALLMAX has identified either a smaller *δ*-set of *S*_*X*_ or, if the traversal is exhausted, *S*_*X*_ itself. In the former case, the procedure stops and returns the visited subset $S^{\prime }_{X}$ of *S*_*X*_. In the latter case, the algorithm continues the search for a smaller *δ*-set in the corresponding other vertex set *S*_*Y*_, *Y*={*G*,*H*}∖*X*, and will return such if found. Otherwise, the smallest maximal *δ*-set in both *S*_*G*_ and *S*_*H*_ is the set itself. This also leads to a disruption of the while-loop (lines 4-12) and, by convention, the return of set $S_{H}^{\prime }$ (=*S*_*H*_).

Because SMALLMAX does not go further than distance *δ* from any already visited node of $S_{X}^{\prime }$, it is clear that the returned vertex set is a *δ*-set. It is also maximal, because the algorithm does not stop prior to having found all vertices of *S*_*X*_ that can be reached from the starting node (which is also a member of *S*_*X*_ and $S_{X}^{\prime }$).

The time complexity of algorithm DECOMPOSE depends on the number of its own recursive function calls. The decomposition of set *S* into sets $S^{\prime }$ and $S\setminus S^{\prime }$ that is performed in line 5 of DECOMPOSE takes *O*(|*S*|) time, but is overshadowed by the time complexity of SMALLMAX. For SMALLMAX, the most costly operation is the search for the next node *s* of $S_{X}\setminus S_{X}^{\prime }$. This can be found through successive traversal of each graph using *breadth-first search* (BFS) outgoing from any arbitrary vertex of sets *S*_*G*_ and *S*_*H*_, respectively. The BFS determines the running time of SMALLMAX and requires *O*(|*V*(*G*)|+|*E*(*G*)|+|*V*(*H*)|+|*E*(*H*)|) time. In the worst case, DECOMPOSE needs $|V_{\cap }|$ iterations to decompose the initial, shared vertex set $V_{\cap }$.

This leads to an overall running time of $O\left (|V_{\cap }| \cdot (|V(G)|\right.\left.+|E(G)|+|V(H)|+|E(H)|)\right)$ for Algorithm 1.

### The special case of shortest-path graphs

In the special case where each pair of vertices *u*, *v* of vertex set $V_{\cap }$ has a directly connecting edge whenever their distance is smaller than or equal to *δ*, SMALLMAX takes $O(|V_{\cap }|)$ time in each iteration. This observation leads to an alternative approach for the general case that may in practice be faster for certain instances or applications: From the input graphs *G* and *H* two new graphs $G^{\prime }$ and $H^{\prime }$ are derived by computing shortest paths between all pairs of vertices in *V*(*G*) and *V*(*H*), respectively. In the new graph $G^{\prime }$ two vertices $u, v \in V(G) = V(G^{\prime })$ are connected with an edge of weight 1 if their distance is smaller than *δ* and, similarly, for graph $H^{\prime }$. Then, the enumeration of *δ*-teams of *G* and *H* is equivalent to computing 1-teams in $G^{\prime }$ and $H^{\prime }$. Our implementation includes an option for the computation of *δ*-teams using this alternative approach. Shortest paths are obtained with Floyd-Warshall’s algorithm which has a running time of $O\left (|V|^{3}\right)$ [20].

### *δ*-teams with families

Family labels allow correspondences between vertices of the input graphs *G* and *H* that go beyond 1-to-1 assignments, which is the scenario best suitable for our application as further explained in “Application to spatial gene cluster discovery”. Given a graph *G*=(*V*,*E*), let $\mathcal F: V \to F$ be a surjective mapping between vertices and families.

We extend the concepts of *δ*-cluster and *δ*-team to families as follows:

#### **Definition 4**

[ *δ*-cluster with families] Given two graphs *G* and *H* with distance measures *d*_*G*_ and *d*_*H*_, respectively, a family mapping $\mathcal F$ and a threshold value *δ*≥0, a pair of vertex sets (*S*_*G*_,*S*_*H*_) with $S_{G} \subseteq V(G)$ and $S_{H} \subseteq V(H)$ is a *δ-cluster* if (i) $\mathcal F(S_{G}) = \mathcal F(S_{H})$ and (ii) *S*_*G*_ and *S*_*H*_ are *δ*-sets in *G* and *H* under distance measures *d*_*G*_ and *d*_*H*_, respectively.

#### **Definition 5**

[ *δ*-team with families] Given two graphs *G* and *H*, a *δ*-cluster (*S*_*G*_,*S*_*H*_) of *G* and *H* is a *δ-team* if it is *maximal*, i.e., there is no *other* *δ*-cluster $(S_{G^{\prime }}, S_{H^{\prime }})$ of *G* and *H* such that $S_{G} \subseteq S_{G^{\prime }}$ and $S_{H} \subseteq S_{H^{\prime }}$.

#### **Example 2**

The two graphs $G^{\prime }$ and $H^{\prime }$ depicted in Fig. *2*b have four *δ*-teams that are in the following represented by their family set: *1*-team {*d*,*f*}; *2*-teams {*c*,*d*,*f*} and {*c*,*e*}, and *3*-team {*a*,*c*,*d*,*f*,*g*}. The set {*c*,*d*,*f*,*g*} exemplifies a non-maximal *3*-cluster of $G^{\prime }$ and $H^{\prime }$.

With the generalization to families, Lemma 1 is no longer applicable. However, Wang et al. [3] provide an adaptation which shows how the original divide-and-conquer approach can be extended:

#### **Lemma 2**

*[3]* Given two graphs *G* and *H*, a family mapping $\mathcal F$ and a threshold value *δ*≥0, let $S_{G} \subseteq V(G)$, $S_{H} \subseteq V(H)$, s.t. $\mathcal F(S_{G}) = \mathcal F(S_{H})$ and *B* be a maximal *δ*-set of *S*_*G*_ or *S*_*H*_. W.l.o.g. let $B \subseteq S_{G}$, then $\text {Teams}_{\delta }(S_{G}, S_{H}) = \text {Teams}_{\delta }(B, S_{H^{\prime }}) \cup \text {Teams}_{\delta }(S_{G}\setminus B, S_{H^{{\prime }{\prime }}})$, where $S_{H^{\prime }} = \{v \in S_{H}\mid \mathcal F(v) \in \mathcal F(B)\}$ and $S_{H^{{\prime }{\prime }}} = \{v \in S_{H} \mid \mathcal F(v) \in \mathcal F(S_{G}\setminus B)\}$.

The adaptations to algorithm DECOMPOSE are a straightforward implementation of Lemma 2 and are shown in Algorithm 3 (DECOMPOSEFAMILIES).



To efficiently retrieve vertices associated with families of $\mathcal F(S^{\prime }_{X})$ and $\mathcal F(S_{X}\setminus S_{X^{\prime }})$ (see lines 6 and 7 of Algorithm 3), we follow Wang et al. [3] and maintain a table of linked lists that maps family identifiers with its members in each respective graph. $\mathcal F\left ({S^{\prime }}_{X}\right)$ can be built in $O\left (\left |{S^{\prime }}_{X}\right |\right)$ time while $\mathcal F\left (S_{X}\setminus {S^{\prime }}_{X}\right)$ needs *O*(|*S*_*X*_|) time. Afterwards, it is possible to build $S^{\prime }_{Y}$ and $S_{Y{^{\prime }{\prime }}}$ in *O*(|*S*_*Y*_|) time. The runtime of SMALLMAX remains the same for Algorithm 3. Yet, because the input sets *S*_*G*_ and *S*_*H*_ can no longer be decomposed into disjoint sets, Algorithm 3 requires overall *O*((|*V*(*G*)|+|*E*(*G*)|)·(|*V*(*H*)|+|*E*(*H*)|)) time and $O\left (|V(G)|+|E(G)|+|V(H)|+|E(H)|\right)$ space.

### Application to spatial gene cluster discovery

We will now demonstrate how the discovery of *δ*-teams with families allows to find spatial gene clusters in genomic data of two or more species. For each genome, we construct an undirected weighted graph in which vertices correspond to genes that are labeled with the identifier of their associated gene family and in which weighted edges correspond to distances obtained from the contact counts of the genomes’ respective Hi-C maps. Then, *δ*-teams (with families according to the genes’ families) in the constructed graphs will correspond to spatial gene cluster candidates.

We first map the Hi-C data onto their chromosomal sequences. In doing so, we associate genes with segments of the Hi-C map. Consequently, contact counts between genes correspond to the contact counts of their associated segments. The value of a contact count does not represent a distance but a closeness score, hence a transformation is needed. We define the *dissimilarity* between two genes *g*_*i*_, *g*_*j*_ associated with Hi-C map *M* as

1 $$ d_{M}(g_{i}, g_{j}) = \left\{ \begin{array}{ll} 0 & \text{if}\,\,g_{i} = g_{j}\\ \max_{k, l}(M_{kl}) + 1 - M_{ij} & \text{otherwise} \end{array}\,.\right.  $$

Note that intrachromosomal distances are symmetric. Whenever two adjacent genes fall into the same segment of an intrachromosomal Hi-C map, the distance is estimated by incorporating their proximity on the DNA sequence. To this end, each base pair between the midpoints of two genes is scored with a relative contact count of *C*/*r*, where *C* is the average contact count between two adjacent segments in the Hi-C map, i.e., the mean of *M*_*i*,*i*+1_ of Hi-C map *M*, and *r* is the resolution of the Hi-C map, i.e., the size of its segments. This estimator works well for our purposes because Hi-C data shows strong correlations with distances on the chromosomal DNA sequence.

It is common that Hi-C maps contain large numbers of empty cells as a result of erroneous measurements and deliberate blanking of the contact counts around the centromere. We do not apply any correction to such cells except to those in intrachomosomal Hi-C maps that correspond to adjacent segments, i.e., the *M*_*i*,*i*+1_ cells. Here, we use the same estimator as described above for genes falling into the same cell of the intrachromosomal Hi-C map.

Because we will compare distances obtained from Hi-C maps of different experiments, we must ensure that they all use the same scale. We do this by normalizing all distances of each Hi-C map *M* as follows:

2 $$ M_{norm} = \frac{c}{\max_{k,l}\left(M_{kl}\right)}\cdot M,  $$

where *c* is the *average maximum contact count* across all Hi-C maps.

### Quantifying functional associations of gene clusters using gene ontology annotations

We quantify functional associations between genes of a gene cluster candidate by testing against the *null* hypothesis that genes in a gene cluster are as functionally associated to their co-members as members within any other equally-sized set of genes in the genome. To this end, we make use of *Gene Ontology* (GO) [21] annotations and relate between gene functions by means of the gene ontology hierarchy that corresponds to the domain “Biological Process”. In doing so, we measure *GO-based functional dissimilarity* (GFD) [22] between pairs of GO-annotated genes. Given a directed acyclic graph *G*=(*V*,*E*) corresponding to a GO-hierarchy, $r_{G}(g) = \left \{ v \in V(G) \mid g \text { associated with } v\right \}$ denotes the set of *GO terms*, i.e., vertices of the GO hierarchy *G*, with which gene *g* is associated. Further, *p*_*G*_(*u*,*v*) denotes the length of the shortest path between two vertices *u*,*v*∈*V* measured in the number of separating nodes. The GFD between two GO-annotated genes *g* and $G^{\prime }$ is then defined as

3 $$ \mathit{gfd}_{G}\left(g, g^{\prime}\right) = \min_{(u, v) \in r_{G}(g) \times r_{G}(g^{\prime})} \left(\frac{p_{G}(u, v)}{\mathit{depth}_{G}(u)+\mathit{depth}_{G}(v)}\right),  $$

where *depth*_*G*_(*w*) is the length of the path from the root vertex of *G* to vertex *w*. This measure gives then rise to the *gene cluster penalty* defined for a gene set $C \subseteq \mathcal G$ of a genome $\mathcal G$ as follows:

4 $$ \phi_{G}(C, \mathcal G) = \sum_{g \in C} \left(\min_{g^{\prime} \in C \setminus \{g\}} \mathit{gfd}\left(g, g^{\prime}\right) - \min_{g^{{\prime}{\prime}} \in \mathcal G \setminus \{g\}} \mathit{gfd}\left(g, g^{{\prime}{\prime}}\right)\right)\,.  $$

This leads to the null hypothesis that the gene cluster penalty of a gene cluster follows the same distribution as any other equally-sized set of genes that is uniformly drawn from the genome. In our analysis, we rank gene clusters according to *p*-values empirically computed from sample pools of size 10^7^ which are drawn for each gene cluster size, individually.

## Results

### The GraphTeams workflow

We implemented Algorithm 3 in the Python programming language and provide an entirely automated Snakemake [23] workflow for the identification of spatial gene clusters. Our workflow, called GraphTeams, takes as input the fully assembled sequences of a collection of genomes as well as their corresponding Hi-C maps. If supplied with Hi-C maps of different resolution, it automatically assimilates their scale. Next, GraphTeams normalizes the Hi-C maps, establishes relationships between Hi-C segments and genes, and constructs weighted graphs that are then input to Algorithm 3. Further, when provided with additional GO-annotations, our workflow allows the computation of a GFD-based ranking scheme for gene cluster candidates. Our approach is, to the best of our knowledge, the first of its kind that is capable of identifying spatial gene clusters. GraphTeams can be obtained from http://github.com/danydoerr/GraphTeams.

### Intrachromosomal study of human and mouse

We used GraphTeams to find intrachromosomal spatial gene cluster candidates in human and mouse. To this end, we supplied GraphTeams with intrachromosomal Hi-C maps first published by Dixon et al. [8]. These Hi-C maps have been obtained from Hi-C sequencing experiments with embryonic cell lines and have a resolution of 40 kb. Further, we queried the Ensemble Genome Browser (release 88) [24] to obtain information about orthologous genes of the human reference sequence GRCh38.p10 and the mouse reference sequence GRCm38.p5. The data consists of 19,843 human genes that are orthologous to 20,647 mouse genes. After integration with the Hi-C data, GraphTeams constructed graphs with average pairwise distances of 408.5 and 407.8 for human and mouse, respectively.

**Fig. 3:**
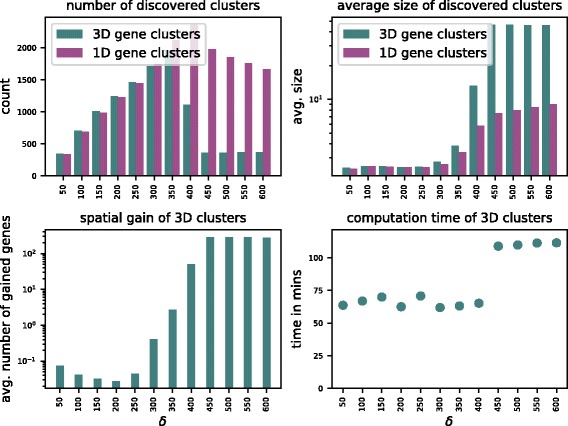
Results of Algorithm 3 on intrachromosomal Hi-C datasets of human and mouse for different values of *δ*. The plots show for each threshold value *δ*, the number of discovered 1D and 3D gene clusters (upper left) and their average sizes (upper right) in the spatial and sequential graphs, respectively, the average number of gained genes in the 3D gene clusters versus the 1D gene clusters (lower left), and the computation time for the 3D gene clusters (lower right)

We ran GraphTeams with a range of values for *δ* from 50 to 600. All computations were performed on a Dell RX815 machine with 64 2.3 GHz AMD Opteron processors and 512 GB of shared memory. The running times for computing all *δ*-teams for each value of *δ* are shown in the bottom right plot of Fig. 3 and range from 62 minutes for *δ*=200 to 111 minutes for *δ*=600. The plot indicates a sharp increase of running time for *δ*>400 that correlates with the increase of the size of identified *δ*-teams in our dataset.

Apart from the performance of our algorithm, we also investigated the claim whether spatial data can improve the search for *functional* gene clusters. Next to the graphs that were generated as previously described and which we will further call *spatial graphs*, we constructed a second type of graphs, called *sequential graphs*. These graphs represent one-dimensional distances between genes if each chromosome is seen as a linear DNA molecule. Because the distances should be comparable to those from spatial graphs, we derived them also from the Hi-C data, but only considered cells *M*_*i*,*i*+1_ of each Hi-C map *M*. The entries directly above the main diagonal of a Hi-C map correspond to contact counts between adjacent segments of a chromosome. This resulted in graphs harboring identical one-dimensional distances as spatial graphs, but without any three-dimensional “shortcuts”. Since our algorithm is a direct generalization of previous methods acting on linear DNA sequences, we use it to identify (traditional) *δ*-teams on sequential graphs, too. We call *δ*-teams that are found in spatial graphs *3D gene clusters*, whereas those in sequential graphs are called *1D gene clusters*.

Figure 3 shows the results for both graphs. In the plot on the top left, we can see that the number of gene clusters grows for both types of graphs with increasing values of *δ* while the number of 3D gene clusters is slightly higher than that of 1D gene clusters. This changes after *δ*=350 when more 3D gene clusters are merged than new instances are found, leading to a rapid decrease in their number along with an increase in their size (see plot on the top right). The peak associated with this phenomenon is delayed in the sequential graphs, owing to the fact that in the latter, gene clusters are more stretched out. This is also the reason why we find that some 1D gene clusters are much denser in the spatial graphs. More surprisingly, we also find gene clusters that can only be found in spatial graphs for a given threshold value *δ*. We call the average amount of genes in a cluster that can be found in the spatial graphs, but not in the sequential ones, *spatial gain* (see plot at the bottom left). We see an increase in spatial gain around *δ*=250 until a saturation seems to be reached at *δ*=450.

We studied in further detail gene clusters that were discovered with *δ*=350. These gene clusters strike a fair balance between number and size as can be readily observed from our previous analysis. The datasets of both, 3D and 1D gene clusters, were used to evaluate functional associations between gene cluster members. To this end, GO-annotations of the human genome were obtained from [21] to compute gene cluster penalties and to rank gene clusters according to their empirical *p*-valueas described in “Quantifying functional associations of gene clusters using gene ontology annotations”. In the obtained gene ontology dataset, 15,737 out of 19,843 human genes were associated with one or more GO-terms. Because the analysis is restricted to those genes with annotated GO-terms, only 1559 out of 1961 3D gene clusters and 1669 out of 2118 1D gene clusters could be further investigated. 18.54*%* of the 3D gene clusters and 18.33*%* of the 1D gene clusters exhibited a significant empirical *p*-value (*p*<0.05). Overall, significant 3D gene clusters tend to include more (annotated) genes (total: 930) than their 1D counterparts (total: 886). Table 1 lists the top twenty 3D gene clusters that are either not found in the set of significant 1D gene clusters, or only partially found, or broken into two or more sub-clusters.

**Table 1:** Top 20 3D gene clusters with smallest *p*-value using intrachromosomal Hi-C data

Name	Genes	Penalty	*p*-Value
HOXC ^∗^	HOTAIR_2, HOTAIR_3, HOXC10, HOXC11, HOXC12, HOXC13, HOXC4, HOXC5, HOXC6, HOXC8, HOXC9	0.006	1·10^−7^
OR	OR5AP2, OR5AR1, OR5M1, OR5M10, OR5M11, OR5M3, OR5M8, OR5M9, OR5R1, OR8K1, OR8U1, OR9G1, OR9G	0	1·10^−7^
IGHV ^∗^	IGHV3-11, IGHV3-13, IGHV3-20, IGHV3-21, IGHV3-23, IGHV3-30, IGHV3-33, IGHV3-35, IGHV3-64D, IGHV3-7	0	1·10^−7^
KRTAP ^∗^	KRTAP13-1, KRTAP13-2, KRTAP13-3, KRTAP13-4, KRTAP15-1, KRTAP24-1, KRTAP26-1, KRTAP27-1	0	1·10^−7^
TAS2R	TAS2R14, TAS2R19, TAS2R20, TAS2R31, TAS2R46, TAS2R50	0	3.70·10^−6^
OR	OR2A12, OR2A14, OR2A25, OR2A5	0	9.09·10^−5^
ZSCAN4	NKAPL, ZKSCAN3, ZKSCAN4, ZSCAN26	0.006	0.00015
TRAV	TRAV12-1, TRAV12-2, TRAV12-3, TRAV13-1, TRAV13-2, TRAV17, TRAV18, TRAV19, TRAV22, TRAV23DV6, TRAV5, TRAV8-1, TRAV8-3, TRAV9-2	0	0.00037
OR	OR5AC1, OR5H1, OR5H14	0	0.00037
IGHV ^+^	IGHV1-18, IGHV1-24, IGHV1-3	0	0.00037
BTN3 ^+^	BTN3A1, BTN3A2, BTN3A3	0	0.00037
*(unnamed)*	GTF2A1L, STON1, STON1-GTF2A1L	0	0.00037
CYP3A	CYP3A4, CYP3A43, CYP3A5, CYP3A7, CYP3A7-CYP3A51P	0.028	0.00037
*(unnamed)*	ADGRE1, C3, CD70, GPR108, TNFSF14, TRIP10, VAV1	0.057	0.00047
ZNF	CCDC106, FIZ1, U2AF2, ZNF524, ZNF580, ZNF784, ZNF865	0.097	0.00110
OR	OR8B12, OR8B4, OR8B8	0.012	0.00376
KIR	KIR2DL1, KIR2DL3, KIR2DL4, KIR2DS4, KIR3DL1, KIR3DL2, KIR3DL3	0.179	0.00243
MMP	MMP12, MMP13, MMP3	0.035	0.00486
TSPY ^+^	TSPYL1, TSPYL4	0	0.00504
SIGLEC ^+^	SIGLEC12, SIGLEC8	0	0.00504

Clusters that can be found as split sub-clusters in the 1D results are marked by an asterisk. Those completely absent in the 1D results are marked by a plus

### Interchromosomal spatial gene cluster candidates in human and mouse

**Fig. 4:**
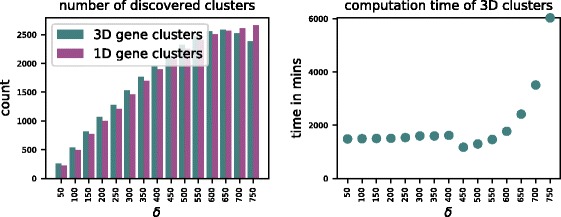
Results of Algorithm 3 on interchromosomal Hi-C datasets of human and mouse for different values of *δ*. The plots show for each threshold value *δ*, the number of discovered 1D and 3D gene clusters in the spatial and sequential graphs, respectively (left) and the computation time for the 3D gene clusters (right)

Unlike traditional methods for gene cluster discovery, our approach is not limited to the study of intrachromosomal clusters. On the contrary, our model permits spatial gene clusters to be composed of genes from different chromosomes. The GraphTeams workflow supports data from both inter- and intrachromosomal Hi-C maps. Here, we present results of an analysis in which we replaced the human Hi-C data from the previous analysis with that by Lieberman-Aiden et al. [9]. The latter consists of both intra- and interchromosomal Hi-C maps and further differs from Dixon et al.’s dataset in the facts that it was obtained from lymphoblastoid cell lines with a lower resolution (1 Mb). Due to the normalization using Eq. 2, distances in the constructed graphs exhibit a wider range than in our previous analysis. This also affects intrachromosomal distances that are now larger than before. On average, two genes have a pairwise distance of 799.8 and 836.6 in human and mouse, respectively. Yet, the number of *δ*-teams in both graphs follows the same trends that we observed in our previous analysis (see left plot of Fig. 4). Thus, following the same line of reasoning, we decided to study gene clusters for *δ*=700 and *δ*=750 in close detail. Note that the larger size of the graphs is taking a toll on the computation time of *δ*-teams (see right plot of Fig. 4). For *δ*=700, GraphTeams reported 58 gene cluster candidates whose members are located on more than one chromosome of the human genome. For *δ*=750, it reported 88, many of which are contained exactly or partially in the former list. Five of these candidates were ranked as significant in our gene ontology analysis as shown in Table 2.

**Table 2:** Interchromosomal gene cluster candidates identified by Algorithm 3 with *δ*=700 and *δ*=750

Name	Genes	*p*-Value
USP17L	*chr. 4*: USP17L10, USP17L11, USP17L12, USP17L13, USP17L15, USP17L17, USP17L18, USP17L19, USP17L20, USP17L21, USP17L22, USP17L23, USP17L24, USP17L25, USP17L26, USP17L27, USP17L28, USP17L29, USP17L30, USP17L5; *chr. 8*: USP17L1, USP17L2, USP17L8	1·10^−7^
OR4F	*chr. 1*: OR4F16, OR4F29; *chr. 8*: OR4F21; *chr. 15*: OR4F15, OR4F6	1.40·10^−5^
GGT	*chr. 20*: GGTLC1; *chr. 22*: GGT2, GGTLC2, GGTLC3	9.09·10^−5^
*OR4M/OR4N*	*chr. 14*: OR4M1, OR4Q3; *chr. 15*: OR4M2, OR4N4, RP11-294C11.1, RP11-294C11.3	9.09·10^−5^
OR4F	*chr. 1*: OR4F5; *chr. 19*: OR4F17	0.0050417
*(unnamed)*	*chr. 20*: RN7SKP271; *chr. 22*: RN7SKP221, RN7SKP63	–
*(unnamed)*	*chr. 1*: RNU1-13P, RNVU1-1, RNVU1-7; *chr. 21*: U1	–
*(unnamed)*	*chr. 3*: RNU6ATAC29P; *chr. 8*: RNU6ATAC41P; *chr. 14*: RNU6ATAC30P; *chr. 17*: RNU6ATAC7P; *chr. 18*: RNU6ATAC20P; *chr. 20*: RNU6ATAC17P, RNU6ATAC34P	–
*(unnamed)*	*chr. 12*: RNU6-101P, RNU6-768P; *chr. 13*: RNU6-68P, RNU6-81P; *chr. 17*: RNU6-450P	–
*(unnamed)*	*chr. 9*: 1193P, RNU6-1269P, RNU6-368P, RNU6-538P, RNU6-599P, RNU6-798P; *chr. 13*: RNU6-55P	–
*(unnamed)*	*chr. 12*: RNU6-1183P; *chr. 14*: RNU6-976P; *chr. 16*: RNU6-758P	–
*(unnamed)*	*chr. 6*: RNU7-48P; *chr. 11*: RNU7-50P; *chr. 13*: RNU7-88P	–
*(unnamed)*	*chr. 5*: RN7SL689P; *chr. 6*: RN7SL502P; *chr. 9*: RN7SL338P; *chr. 10*: RN7SL518P; *chr. 12*: RN7SL519P; *chr. 13*: RN7SL597P; *chr. 15*: RN7SL497P; *chr. 17*: RN7SL138P; *chr. 20*: RN7SL116P; *chr. 22*: RN7SL704P	–

The upper part shows all clusters that received a significant *p*-value in the GO analysis. The lower part lists clusters containing no GO-annotated genes, but were identified by manual inspection as corresponding to associated gene families

## Discussion

The enumeration of common intervals in sequences has been subject to various extensions including *δ*-teams. Here, we described a generalization of *δ*-teams from sequences to graphs. We presented a novel algorithm for the enumeration of *δ*-teams that, when trivially extended to *k* graphs $G_{i} = \left (V_{i}, E_{i}\right)$, for *i*=1,…,*k*, will run in $O\left (\sum _{i} |V_{i}| + |V_{\cap }|\cdot \sum _{i} \left (|V_{i}|+|E_{i}|\right)\right)$ time and $O\left (\sum _{i}(|V_{i}|+|E_{i}|)\right)$ space, where $V_{\cap } = V_{1} \cap \cdots \cap V_{k}$. Our algorithm beats the naive approach that requires $O\left (\sum _{i}|V_{i}|^{3}\right)$ time and $O\left (\sum _{i}|V_{i}|^{2}\right)$ space. The naive approach computes all-pairs shortest paths on each of the input graphs independently and then employs a standard single-linkage algorithm for the enumeration of *δ*-teams in a matrix over all largest shortest paths of all graphs.

Further, we provide an algorithm for the computation of *δ*-teams that, when trivially extended to *k* graphs *with families*, will run in $O\left (k \cdot \prod _{i} \left (|V_{i}|+|E_{i}|\right)\right)$ time and $O\left (k\cdot \sum _{i}\left (|V_{i}|+|E_{i}|\right)\right)$ space. In comparison, the best algorithm for the enumeration of *δ*-teams in *k* permutations of size *n* runs in $O\left (k \cdot n \cdot \log N\right)$ time, where *N* denotes the number of reported *δ*-teams [25]. The best algorithm that solves the corresponding family-based problem for *k* sequences of lengths *n*_1_,…,*n*_*k*_ runs in $O\left (k \cdot C \cdot \log \left (n_{1}\cdots n_{k}\right)\right)$ time, where *C* is a factor accounting for the number of possible 1:1 assignments between family members across the *k* graphs [3]. The differences in running time between the permutation-, sequence- and our graph-based algorithms reflect the fact that the latter solve much more general problems.

With GraphTeams, we developed an *open source*, fully automated workflow for gene cluster discovery in Hi-C data. We used this workflow to study intrachromosomal gene clusters in a Hi-C dataset of embryonic cell lines from human and mouse [8]. In doing so, we assessed the benefit of predicting gene clusters in spatial data as opposed to traditional, sequential genomic data. We identified several gene cluster candidates corresponding to sets of functionally associated genes whose members are closer to each other in the 3D space than on the chromosomal sequence (see Table 1). Many of these gene clusters are already known from the literature. E.g., we find four clusters of *olfactory receptor* (OR) genes on different chromosomes, the *taste receptor type 2* (TAS2R) gene cluster and the HOXC gene cluster. The latter is one of three clusters among the top 20 but can be found in the 1D results only as a composition of sub-clusters. Therefore, these genes seem to be even closer together in 3D space than on the DNA strand. The same is true for other clusters, such as that of the *testis-specific protein Y-encoded* (TSPY) and *superfamily Ig belonging lectins* (SIGLEC) which were not even partially detected in the 1D graphs.

We then extended our approach to the discovery of interchromosomal gene clusters and applied it to a mixed dataset containing Hi-C data from a human lymphoblastoid cell line [9] and the previously studied intrachromosomal data of an embryonic cell line of the mouse. Table 2 lists several identified gene cluster candidates that contain genes located on different chromosomes in the human genome. The highest ranking cluster constitutes 23 out of 115 members of the human USP17L gene family. Since the divergence from the common ancestor of human and mouse, this family of deubiquitinating enzymes has largely expanded in the human lineage and is homologous to only four genes located on chromosome 7 in the mouse. We further identified three gene cluster candidates related to olfactory receptors and one related to members of the gamma-glutamyltransferase (GGT) gene family. The vast majority of the studied gene cluster candidates does not contain GO-annotated genes. Further examination revealed that many of these clusters are entirely composed of genes encoding small nuclear RNAs. The lower part of Table 2 lists the eight most promising gene cluster candidates identified through manual inspection.

## Conclusions

By identifying *δ*-teams *with families*, we provide a flexible model that is well suitable to capture the complexity of biological datasets such as those at hand. Our analysis of Hi-C data from human and mouse reveals several known gene clusters (thus validating our approach), but also few sparsely studied or possibly unknown gene cluster candidates that could be the source of further experimental investigation.

The presented algorithms and their implementation are fast enough to process large graphs as demonstrated in a study of Hi-C data of human and mouse. Nevertheless, further research may lead to improved algorithms. It seems possible that the problem of finding *δ*-teams in graphs *without families* could be solved faster with the help of a guide tree that allows to find a maximal *δ*-set by traversing each graph in fewer steps than required by an exhaustive graph traversal. Alternatively, a randomized variant of our algorithm could assert a better expected running time. The presented algorithmic work could also be extended into another direction, by allowing the direct computation of the *single-linkage hierarchy*. This makes the gene cluster analysis no longer dependent on a fixed *δ*, but will provide all possible *δ*-clusters through a single computation. This idea has also been applied for *δ*-teams in sequences, where the hierarchy is called *gene team tree* [4,26].

The *δ*-team model has the drawback that only maximal *δ*-clusters are reported. These can potentially hide smaller, nested *δ*-clusters that are in size closer to those of typical gene clusters. However, the solution space of non-maximal *δ*-clusters in graphs is exponential, which leaves little hope for their efficient enumeration and subsequent processing.
